# Supplementary figures and images for: Identification of Three Novel Conidiogenesis-Related Genes in the Nematode-Trapping Fungus Arthrobotrys oligospora
Source: Pathogens. 2022 Jun 23;11(7):717. doi: 10.3390/pathogens11070717 (PMC9324328; doi:10.3390/pathogens11070717)

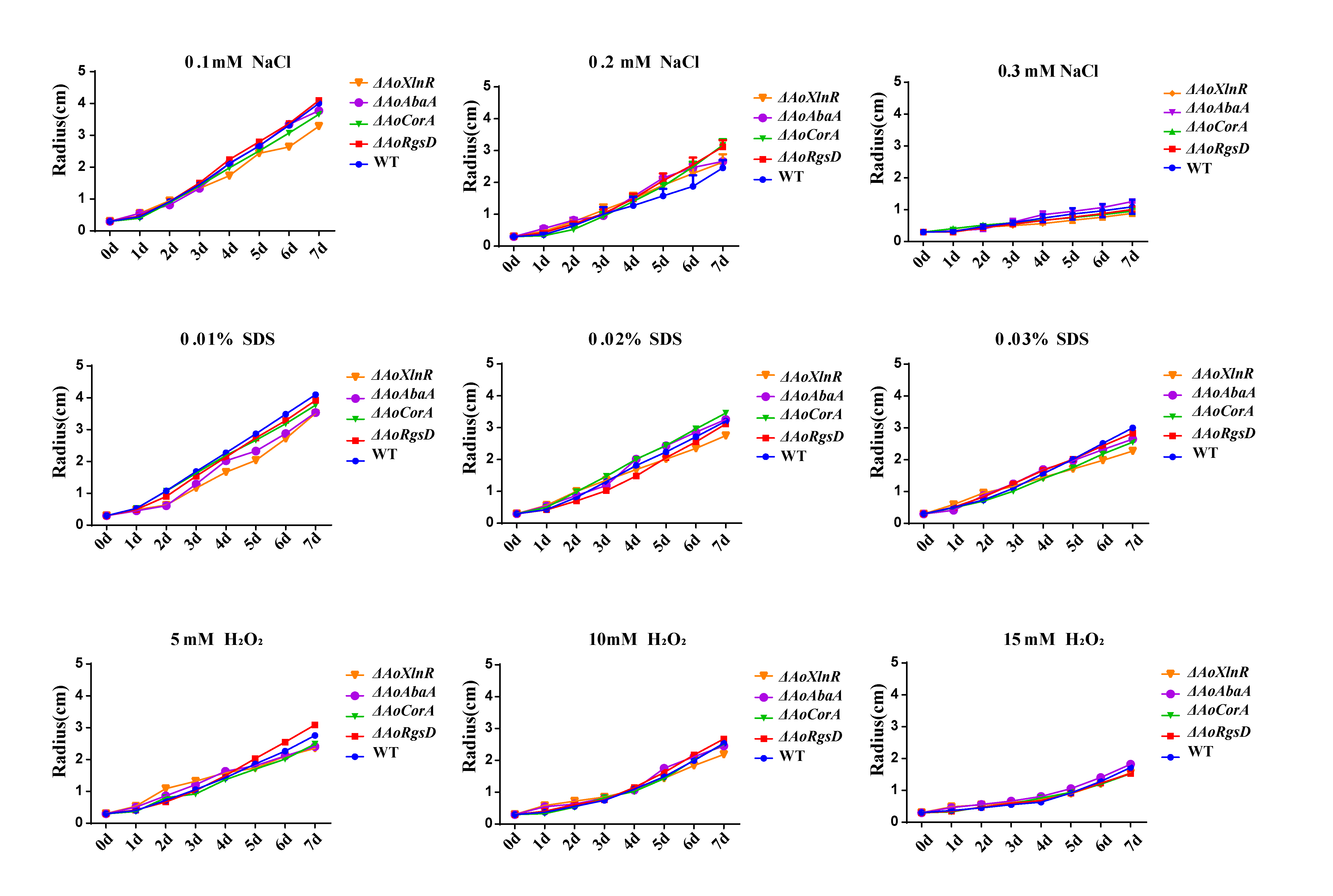

Supplement: Supplementary file 1 [file pathogens-11-00717-s001.zip › Figure S1.tif]

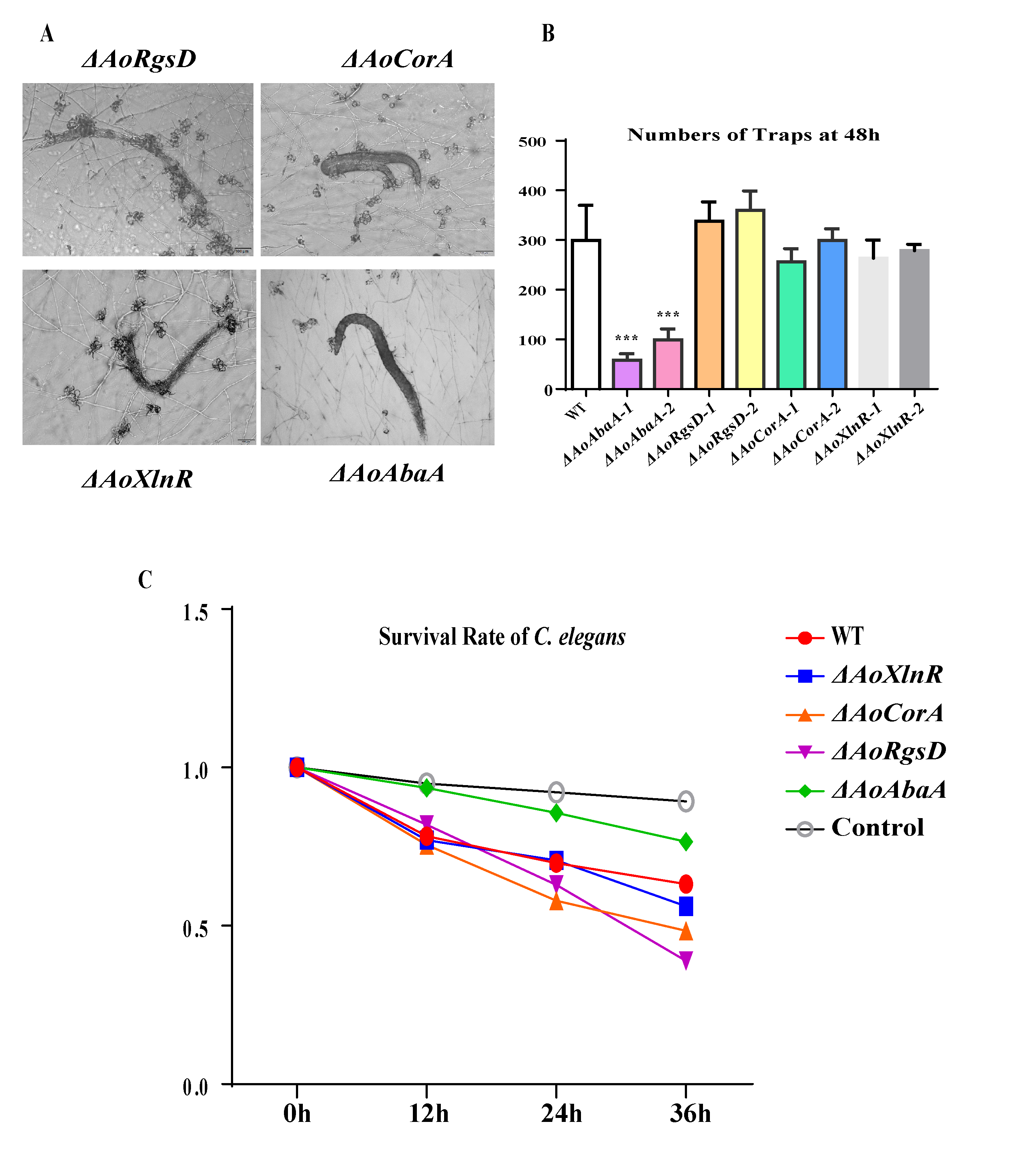

Supplement: Supplementary file 1 [file pathogens-11-00717-s001.zip › figure S2 .tif]

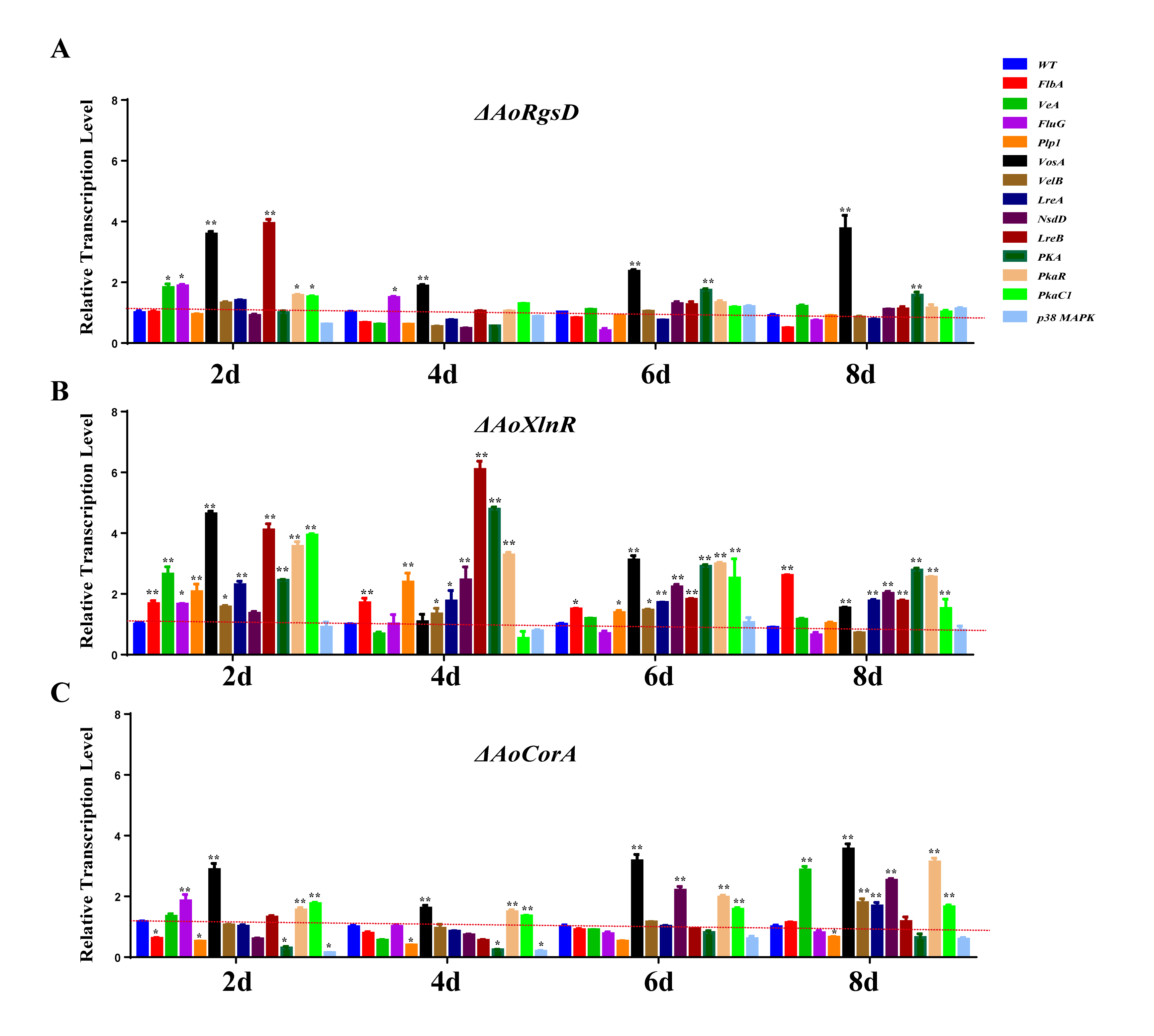

Supplement: Supplementary file 1 [file pathogens-11-00717-s001.zip › figure S3 .tif]
